# Immune checkpoint inhibitor-induced inflammatory arthritis with severe joint destruction requiring knee arthroplasty

**DOI:** 10.1093/rap/rkaf067

**Published:** 2025-05-30

**Authors:** Hirokazu Taguchi, Yoshitaka Ueda, Yuichi Nagase, Naoto Yokogawa

**Affiliations:** Department of Rheumatic Diseases, Tokyo Metropolitan Tama Medical Center, Fuchu, Japan; Department of Rheumatic Diseases, Tokyo Metropolitan Tama Medical Center, Fuchu, Japan; Department of Rheumatic Surgery, Tokyo Metropolitan Tama Medical Center, Fuchu, Japan; Department of Rheumatic Diseases, Tokyo Metropolitan Tama Medical Center, Fuchu, Japan

Key message

- Early recognition of immune checkpoint inhibitor–induced inflammatory arthritis is important to prevent joint destruction.

Dear Editor, Inflammatory arthritis (IA) occurs as an immune-related adverse event (irAE) in 1–7% of patients receiving immune checkpoint inhibitor therapy [1]. irAE-induced IA (irAE-IA) is often overlooked because it has a later onset than other irAEs and is not life-threatening [2]. A previous, single-centre, retrospective study reported a mean duration of 5.2 months from irAE-IA symptom onset to diagnosis, which was longer than that for other irAEs [2]. More than half of IA cases progress to chronic arthritis [3]. A previous report briefly described the case of an irAE-IA patient in whom severe joint destruction developed 1 year after irAE-IA was diagnosed; the patient later underwent arthroplasty [4]. However, no reports have as yet provided a detailed description of the clinical course of irAE-IA with joint destruction.

A 69-year-old male patient was referred to our department for a 4-month history of right knee arthralgia. Fourteen months earlier, the patient had received a diagnosis of stage IV lung adenocarcinoma and had begun pembrolizumab therapy. He later experienced an irAE-associated skin rash, hypothyroidism and adrenocorticotropic hormone deficiency. A physical examination revealed right knee arthritis. His serum CRP level was 4.32 mg/dl (reference 0.00–0.14 mg/dl). The test results for RF, anti-CCP antibody and ANA were negative. Synovial fluid analysis of the right knee joint demonstrated a white blood cell count of 44 400 cells/μl, with 94.9% mononuclear cells and 5.1% polynuclear cells. Neither monosodium urate nor calcium pyrophosphate crystals were detected in the synovial fluid and a bacterial culture was negative. Musculoskeletal ultrasound (MSUS) of the right knee revealed synovitis in the suprapatellar bursa (Fig. 1A) and bone erosion. Radiography of the knee joints in the weight-bearing position found joint space narrowing only in the right knee joint (Fig. 1B, C). Although the involvement of OA could not be completely ruled out, the asymmetry in joint destruction and the MSUS findings supported a diagnosis of irAE-IA with severe joint destruction. Oral prednisolone (PSL) at 20 mg/day and sulfasalazine were administered, leading to the normalization of serum CRP. Methotrexate was avoided because of a past medical history of liver cirrhosis. Although sulfasalazine carries a risk of hypersensitivity in patients with irAE-IA, it was administered to facilitate the tapering of PSL. To minimize the adverse effects, PSL was rapidly tapered to 5 mg/day. Throughout PSL tapering, serum CRP levels remained low and MSUS findings showed improvement in synovitis. However, the right knee joint pain persisted and the patient continued to require a cane for ambulation. Therefore, 6 months after the diagnosis, the patient underwent a right total knee arthroplasty. Macroscopically, proliferative reddish synovium was observed in the suprapatellar bursa (Fig. 1D). Histological analysis of the synovium confirmed the presence of proliferative synovium and inflammatory cell infiltration composed predominantly of CD4^+^ and CD8^−^ lymphocytes (Fig. 1E–G). Postoperatively, the patient’s joint pain improved, and at postoperative month 2, subcutaneous adalimumab therapy was begun to control the arthritis.

**Figure 1.**
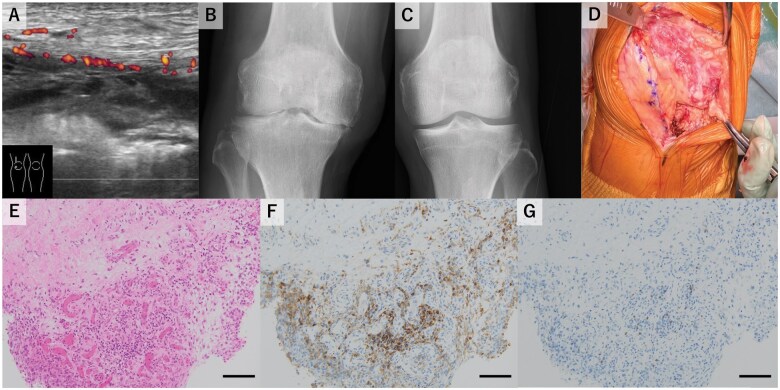
irAE-IA with joint destruction. (**A**) Synovitis in the suprapatellar bursa on the MSUS. Joint space narrowing in the (**B**) right and (**C**) left knee joints on radiography. (**D**) Intra-operative findings demonstrating proliferative, reddish synovium in the suprapatellar bursa. (**E**) Histological findings of the right knee synovium (haematoxylin and eosin staining; original magnification × 200; scale bar: 100 μm). (**F**) CD4 and (**G**) CD8 staining of the right knee synovium (original magnification × 200; scale bar: 100 μm).

irAE-IA is a form of IA caused by activated T cells in the synovial tissue. Like RA, it follows a chronic course; however, few studies have reported joint-destructive changes associated with this condition. A previous single-centre, prospective, observational study of irAE-IA found joint bone erosion by imaging studies in 5 of 46 patients [5]. Another study reporting MSUS findings in nine irAE-IA patients and found bone erosions in three patients. Notably, in two of these patients, the erosion was observed at an early stage of the disease (months 0 and 2 after onset, respectively) [6]. Single-cell transcriptomic and antigen receptor repertoire analyses of the synovial fluid demonstrated significantly more CD8^+^ cytotoxic T cells in irAE-IA than in RA or PsA [4], suggesting that irAE-IA has a potentially higher risk of joint destruction. Notably, irAE-IA is often diagnosed late [2], and studies have indicated that the severity of the arthritis correlates with the time to diagnosis [5]. These facts suggest that joint destruction in the present patient might have begun at an early stage of the irAE-IA. On the other hand, in the present case, CD8^+^ T cells were absent in the synovium. In a previous biopsy case of irAE-IA, CD4^+^ T cells were observed predominantly in synovial tissue, as in the present case [7]. Thus the histopathological features of irAE-IA suggest diversity, and histopathological stratification, such as that in RA, may help predict prognosis, including the risk of joint destruction [8].

In summary, the present report described the details of the clinical course of irAE-IA requiring arthroplasty for severe joint destruction. Early recognition of irAE-IA is crucial to preventing joint destruction.

## Data Availability

Data sharing does not apply to this report, as no datasets were generated for analysis in the present study.
